# Effects of enteral nutrition on heart function, inflammatory markers and immune function in elderly patients with chronic heart failure

**DOI:** 10.12669/pjms.38.1.4451

**Published:** 2022

**Authors:** Dan Zhang, Hongli Li, Xiang Tian, Sujuan Zhang

**Affiliations:** 1 Dan Zhang, Department of Outpatient, Baoding First Central Hospital, Baoding, Hebei 071000, China; 2 Hongli Li, Department of Cardio-vascular Baoding First Central Hospital, Baoding, Hebei 071000, China; 3 Xiang Tian, Department of Cardio-vascular Baoding First Central Hospital, Baoding, Hebei 071000, China; 4 Sujuan Zhang Department of Outpatient, Baoding First Central Hospital, Baoding, Hebei 071000, China

**Keywords:** Enteral nutrition, Elderly, Chronic heart failure, Heart function, Inflammatory factors, Immune function, Nutrition

## Abstract

**Objectives:**

To evaluate the effect of enteral nutrition on heart function, inflammatory markers and immune function in elderly patients with chronic heart failure and its clinical significance.

**Methods:**

Eighty patients with moderate and severe heart failure admitted to the Cardiology Intensive Care Unit (CCU) of Baoding First Central Hospital from May 2019 to May 2020 were included in this study and randomly divided into two groups: the experimental group and the control group, with 40 patients in each group. The experimental group was given enteral nutrition support therapy on the basis of conventional therapy for one month, while the control group was given restricted salt and water intake on the basis of conventional therapy, and patients were given free diet according to their wishes. The changes in heart function before and after treatment, changes in inflammatory factors such as TNF-a, CRP, IL-6, changes in levels of immunoglobulins such as IgA, IgM, and IgG, and the improvement of the performance status of the two groups were compared and analyzed.

**Results:**

After treatment, indicators such as BNP, LVEDD, LVEF and 6min walking distance in the experimental group were significantly improved compared with the control group, with statistically significant differences (p<0.05), and the levels of inflammatory factors such as TNF-a, CRP and IL-6 in the experimental group were significantly reduced compared with those in the control group (p=0.00). The levels of IgG, IgA, IgM and other immunoglobulins in the experimental group improved more significantly after treatment than those in the control group, with statistically significant differences (IgG, IgA, p=0.00; IgM, p=0.01). Moreover, the experimental group was significantly superior to the control group in the improvement rate of performance status score (ECOG) after treatment (p=0.04); The incidence of gastrointestinal adverse reactions in the experimental group was 20%, and that in the control group was 15%. No statistically significant difference can be observed in the gastrointestinal tolerance of both groups (p=0.56).

**Conclusions:**

Reasonable enteral nutrition boasts a variety of benefits for the recovery of elderly patients with chronic heart failure. With reasonable enteral nutrition, the heart function of elderly patients with chronic heart failure can be significantly improved, inflammatory factors can be reduced, immunity and performance status can be enhanced, and gastrointestinal tolerance can be ameliorated without obvious gastrointestinal reactions.

## INTRODUCTION

Chronic Heart Failure (CHF), as the final stage of the progression of a variety of cardiovascular diseases, is a clinical syndrome caused by changes in heart structure or function, decreased cardiac output, increased systemic or pulmonary venous pressure, and congestion due to various reasons.^1^ Adverse effects may be caused to patients with CHF due to chronic congestion of pulmonary and systemic circulation. Patients with mucosal congestion in the gastrointestinal tract may suffer from nausea, anorexia, decreased appetite, inability to eat, etc., and malnutrition and a negative nitrogen balance may occur with the progression of the disease course, affecting the quality of life of patients.^2^ Patients with long-term pulmonary congestion may suffer from impaired alveolar respiratory membrane function, resulting in insufficient oxygen diffusion and dyspnea.^3^ These conditions are particularly common in elderly patients. It was believed by Jones et al.^4^ that the prognosis of patients with CHF has a close bearing on their nutritional status. At the same time, it is considered in the study by Wandrag et al.^5^ that malnutrition interacts with CHF. Chronic malnutrition is expected to result in an inadequate supply of energy to the heart muscle and exacerbates heart failure, which in turn leads to further malnutrition. Based on this, Rahman et al.^6^ believed that simple control of heart failure could not achieve ideal results, and the study results showed that^7^ nutritional supports enable patients with CHF to obtain nutrition to control their heart failure. Enteral nutrition is a low-cost and easy-to-accept treatment regimen that provides nutrition via catheter or oral administration, which is in line with human physiological state. With such a treatment regimen, the intestinal function of patients can be improved and certain adverse reactions can be prevented.^8^ Enteral nutrition support therapy combined with conventional therapy is a potent regimen for the treatment of elderly patients with chronic heart failure, which not only has certain benefits on heart function and performance status of patients, but also can improve the level of inflammatory factors and immune function of patients.

## METHODS

Eighty patients with moderate and severe heart failure admitted to the Cardiology Intensive Care Unit (CCU) of Baoding First Central Hospital from May 2019 to May 2020 were recruited and randomly divided into two groups, with 40 cases in each group. 19 males and 21 females were divided into the experimental group, aged 60-75 years old, with an average of 68.89±4.46 years old. 22 males and 18 females were divided into the control group, aged 63-73 years old, with an average of 68.05±3.10 years old. There were no significant differences in the general information of the two groups, which were comparable (Table-I).

**Table-I:** Comparative analysis of general data between the experimental group and the control group (x̄±S) n=40.

Indicators	Experimental group	Control group	t/χ^2^	p
Age	68.89±4.46	68.05±3.10	0.98	0.33
Male (%)	19 (47.5%)	22 (55%)	0.45	0.50
Course of disease (year)	3.73±0.76	3.90±0.87	0.93	0.35
***Etiology***				
Coronary heart disease (case %)	18 (45%)	20 (50%)	0.20	0.65
Pulmonary heart disease (case %)	10 (25%)	13 (32.5%)	0.55	0.46
Cardiomyopathy (case %)	7 (17.5%)	5 (12.5%)	0.39	0.53
Valvulopathy (case %)	5 (12.5%)	2 (5%)	1.41	0.24
***Heart function classification***				
Class III (cases %)	24 (60%)	22 (55%)	0.20	0.65
Class IV (cases %)	16 (40%)	18 (45%)	0.22	0.43
***Complicated underlying disease***				
Hypertension (case %)	15 (37.5%)	18 (45%)	0.46	0.50
Diabetes (case %)	17 (42.5%)	12 (30%)	1.35	0.24
Other (case %)	8 (20%)	10 (25%)	0.28	0.60
NRS score	4.37±0.43	4.28±0.32	1.06	0.29

p>0.05.

### Ethical approval:

The study was approved by the Institutional Ethics Committee of Baoding First Central Hospital (No.:2019048; date: June 18, 2020), and written informed consent was obtained from all participants.

### Inclusion criteria:

1. Patients ≥60 years old;
2. Patients with a history of cardiovascular disease;
3. Patients with heart function of Class III and IV^9^;
4. Patients with typical symptoms of cardiac insufficiency^10^;
5. Patients with left ventricular ejection fraction (LVEF) <40%;
6. Patients with a nutritional risk screening scale (NRS) score ≥3 points^11^;
7. Patients whose family members had signed the consent form and were able to cooperate with the completion of the study.

### Exclusion criteria:

1. Patients with metabolic diseases or chronic wasting diseases such as tumors, autoimmune diseases, chronic inflammatory diseases, hyperthyroidism, etc.;
2. Patients with congenital heart disease or arrhythmia-related diseases;
3. Patients with severe dysfunction of vital organs, such as liver and kidney dysfunction, blood system diseases, which cannot be improved after active correction;
4. Patients who had recently received nutritional therapy;
5. Patients with acute myocardial infarction or cardiogenic shock;
6. Patients who cannot tolerate enteral nutrition or have serious complications;
7. Patients who had recently taken relevant drugs that affect the study such as hormones and immunosuppressant’s.

### Enteral nutrition therapy:

The conventional treatment regimens for patients in the two groups included cardiac agents, diuresis, vasodilation to reduce cardiac preload and afterload,^12^ oxygen inhalation, sedation and pain relief, etc.

The experimental group was given enteral nutrition support therapy on the basis of conventional therapy. Intact protein enteral nutrition was mainly used as nutrition agent, which is rich in trace elements. Dosage was adapted according to gastrointestinal tolerance and dietary fiber was added to promote gastrointestinal function. For patients with diabetes, abundant nutritional dosage forms such as monounsaturated fatty acids and fructose were added.^13^ Patients were given a liquid or semi-liquid diet when necessary according to their wishes, with the minimally invasive energy of about 20 kcal/kg/d, and oral or nasal feeding was adopted according to the wishes of patients. One month was seen as a course of treatment. The control group was given restricted salt and water intake on the basis of conventional therapy, and patients were given free diet according to their wishes.

### Observation indicators:

Comparative analysis of heart function changes: N-terminal brain natriuretic peptide (BNP) was drawn from the fasting venous blood test group before and one month after treatment, and left ventricular ejection fraction (LVEF), left ventricular end diastolic diameter (LVEDD) and 6min walking distance of the patients were detected, respectively..

Inflammatory factor indicators: Periphereal venous blood was drawn from all patients at the basal state in the morning before and two weeks after treatment, respectively. Enzyme-linked immunosorbent assay (ELISA) was utilized to detect the levels of inflammatory factors such as tumor necrosis factor (TNF-a), C-reactive protein (CRP) and interleukin-6 (IL-6).

Immune function indicators: Peripheral venous blood was drawn from the patients at the basal state in the morning before and 2 weeks after treatment, respectively, and immunoglobulins such as IgA, IgM and IgG of the patients were detected to analyze the differences in their changes.

Improvement of performance status: ECOG score^14^ was used to observe the improvement of performance status before and 1 month after treatment: improvement (≥ 1 point reduction), stability (score unchanged), deterioration (≥ 1 point increase).

### Statistical analysis:

All the data were statistically analyzed by SPSS 20.0 software, and the measurement data were expressed as (x ¯±S). Two independent sample t-test was used for inter-group data analysis, repeated measurement analysis of variance was used for intra-group data analysis, and χ^2^ was adopted for rate comparison. P<0.05 indicates a statistically significant difference.

## RESULTS

The changes of heart function indicators of the two groups before and after treatment are shown in Table-II, suggesting that there was no significant difference in BNP, LVEDD, LVEF and 6min walking distance between the two groups before treatment (p>0.05). The above indicators were improved significantly after one month after treatment compared with those before treatment, with a statistically significant difference (p<0.05). The improvement was specifically manifested as a decrease in BNP and LVEDD and an increase in LVEF and six minitues walking distance compared with those before treatment. The experimental group showed a more significant improvement than the control group in the indicators after treatment, with a statistically significant difference (p<0.05).

**Table-II:** Comparative analysis of heart function indicators between the two groups (x̄±S) n=40.

Indicators		Before treatment*	1 month after treatmentΔ	t	p
BNP (pg/ml)	Experimental groupΔ	321.32±35.27	143.42±22.79	26.79	0.00
	Control groupΔ	328.25±36.14	225.73±23.76	14.99	0.00
	t	0.87	15.81		
	p	0.38	0.00		
>LVEF (%)	Experimental groupΔ	41.68±3.67	53.74±4.58	13.00	0.00
	Control groupΔ	42.05±4.21	46.55±5.07	4.32	0.00
	t	0.42	6.55		
	p	0.67	0.00		
LVEDD (mm)	Experimental groupΔ	59.36±3.28	51.23±3.84	10.18	0.00
	Control groupΔ	59.03±3.57	53.65±3.97	6.37	0.00
	t	0.43	2.77		
	p	0.67	0.00		
6min walking distance (m)	Experimental groupΔ	346.53±56.83	385.62±25.83	3.96	0.00
	Control groupΔ	343.79±64.20	369.35±27.14	1.23	0.02
	t	0.20	2.74		
	p	0.84	0.01		

* p>0.05, Δp<0.05.

The changes of inflammatory factors in the two groups of patients before and after treatment are shown in Table-III, suggesting that there was no significant difference in the levels of inflammatory factors such as TNF-a, CRP and IL-6 between the two groups before treatment (p>0.05). The above indexes were lower after treatment than those before treatment, with a statistically significant difference (p<0.05), while that in the experimental group were significantly lower than those in the control group after treatment, with a statistically significant difference (p=0.00).

**Table-III:** Comparative analysis of changes in inflammatory factors before and after treatment between the two groups (x̄±S) n=40.

Group		Before treatment*	2 weeks after treatmentΔ	t	p
TNF-ɑ (ng/L)	Experimental groupΔ	46.03±7.24	21.31±5.31	17.43	0.00
	Control groupΔ	46.25±7.01	27.69±5.72	12.97	0.00
	t	0.14	5.17		
	p	0.89	0.00		
CRP(mg/L)	Experimental groupΔ	82.64±16.32	14.74±4.50	25.37	0.00
	Control groupΔ	81.89±16.71	20.28±4.42	22.54	0.00
	t	0.20	5.55		
	p	0.84	0.00		
IL-6 (ng/L)	Experimental groupΔ	18.43±6.37	8.47±2.26	9.32	0.00
	Control groupΔ	18.35±6.08	12.58±3.06	5.36	0.00
	t	0.06	6.83		
	p	0.95	0.00		

* p>0.05, Δp<0.05.

The immunoglobulin levels in both groups were improved after treatment compared with those before treatment (p=0.00). The experimental group showed a more significant improvement after treatment than the control group, with a statistically significant difference (IgG, IgA, p=0.00; IgM, p=0.01) (Table-IV).

**Table-IV:** Comparative analysis of immunoglobulin levels before and after treatment between the two groups (x̄±S) n=40.

Group		Before treatment*	2 weeks after treatmentΔ	t	p
IgG (g/L)	Experimental groupΔ	7.57±2.36	13.77±4.35	7.92	0.00
	Control groupΔ	7.73±2.27	11.25±4.46	4.45	0.00
	t	0.31	2.56		
	p	0.76	0.01		
IgA (g/L)	Experimental groupΔ	1.35±0.27	2.83±0.76	11.61	0.00
	Control groupΔ	1.32±0.31	2.24±0.42	11.15	0.00
	t	0.46	4.30		
	p	0.65	0.00		
IgM (g/L)	Experimental groupΔ	1.38±0.65	2.75±0.63	9.57	0.00
	Control groupΔ	1.36±0.73	2.39±0.50	7.36	0.00
	t	0.13	2.83		
	p	0.89	0.01		

* p>0.05, Δp<0.05.

The improvement rate of performance status score (ECOG) in the experimental group after treatment was significantly higher than that in the control group (p=0.04), and the experimental group showed a more significant improvement in the performance status of patients (Table-V).

**Table-V:** Comparative analysis of performance status scores (ECOG) before and after treatment between the two groups (X̄±S) n=40.

Group	Improvement*	Stable	Deterioration
Experimental group	23	10	7
Control group	14	18	8
χ^2^	4.07	3.51	0.08
p	0.04	0.06	0.78

* p<0.05.

The incidence of gastrointestinal adverse reactions in the experimental group within 2 weeks of treatment was 20%, while that in the control group was 15%. No statistically significant difference can be observed in the gastrointestinal tolerance between the two groups (p=0.56) (Table-VI).

**Table-VI:** Comparative analysis of gastrointestinal tolerance between the two groups after treatment (X̄ ±S) n=40.

Group	Abdominal distension	Diarrhea	Constipation	Gastric retention	Total	Incidence
Experimental group	4	2	0	2	8	20%
Control group	2	0	3	1	6	15%
χ^2^						0.35
p						0.56

p>0.05.

## DISCUSSION

Chronic lung disease and cardiovascular disease are experiencing an increase in their incidence year by year in the wake of the changes in people’s living habits and surrounding environment.^15^ This is accompanied by prolonged heart damage and remodeling of the myocardial wall, which eventually leads to heart failure.^16^ It was shown in the study by Chung ML et al.^17^ that nutritional abnormalities exert a critical role in the development of CHF. In case of nutritional deficiency, abnormal metabolism of myocardial energy may be caused, which accelerates myocardial remodeling and increases myocardial ischemia and hypoxia. Subsequently, the degree of heart failure will be aggravated, the heart pumping function will be reduced, the pulmonary circulation and systemic circulation congestion will be increased, and the gastrointestinal burden will be increased, eventually leading to clinical symptoms such as anorexia, nausea and reduced eating. Therefore, most patients with CHF are affected by malnutrition. Nutritional therapy plays a definite role in the treatment of chronic heart failure, and nutritional support is becoming the pillar of comprehensive treatment for patients with chronic heart failure.^18^

Rational nutritional support therapy is of great importance to improve heart function and maintain normal physiological metabolism of the body. Studies have shown that^19^ the progression of chronic heart failure can be partially attributed to the deficiency of sugar, protein, fat and trace elements. Induced by leptin in adipose tissue, appetite will decrease with the consumption of body fat storage. Patients suffer from heart failure are usually accompanied by gastrointestinal malabsorption, lipid malabsorption, and even, in severe cases, cardiac cachexia in elderly patients.^20^ The deficiency of protein can directly lead to the contractile function of cardiomyocytes and accelerate their death.^21^ In view of this, a more individualized nutritional therapy is preferred for patients with CHF.

An important role is played by the inflammatory response in patients with chronic heart failure, which can directly lead to the aggravation of abnormal cardiac hemodynamics and the remodeling of the ventricular wall.^22^ TNF-α blocks the release of calcium ions from the sarcoplasmic reticulum and decreases the level of calcium ions in cardiomyocytes, thereby significantly reducing the contractility of cardiomyocytes,^23^ while IL-6, TNF-α and other inflammatory factors have negative inotectonic effects.^24^ It was discovered by Kanda et al. that the expression of IL-6 was increased in the blood circulation and myocardium of patients with heart failure, and the level of IL-6 in the blood circulation was closely linked to the severity of heart failure, which could predict the occurrence of clinical endpoint events of heart failure.^25^ Enteral nutrition can play its unique role not only in substantially alleviating intestinal mucosal damage caused by intestinal ischemia-reperfusion injury, but also in promoting the restoration of the intestinal barrier and reducing the levels of circulating endotoxin, TNF-α and IL-6. It was also confirmed in this study that the levels of inflammatory factors such as TNF-a, CRP and IL-6 in the enteral nutrition group were significantly lower than those in the control group after treatment, with a statistically significant difference (p=0.00).

Enteral nutrition boasts the ability to improve the nutritional status and immune function of patients, thereby promoting the recovery of patients.^26^ It was considered in the study by Arutiunov et al.^27^ that patients with NYHA Class III-IV chronic heart failure in the experimental group who received formal enteral nutrition intervention had significant performance recovery compared with the control group, and the results of 6min walking test were increased by 46.8% and 10.3%, respectively. Arutiunov et al.^28^ believed that nutritional support had certain advantages in improving the general state of CHF patients with any degree of malnutrition. It has also been confirmed in our study that various indicators such as BNP, LVEDD, LVEF and 6-min walking distance in the experimental group after treatment have improved significantly compared with the control group, with statistically significant differences (p<0.05). In addition, the levels of immunoglobulins such as IgG, IgA, and IgM in the experimental group improved more significantly than those in the control group after treatment, with statistically significant differences (IgG, IgA, p=0.00; IgM, p=0.01); The experimental group had obvious advantages over the control group in the improvement rate of performance status score (ECOG) (p=0.04).

### Limitations of the study:

Fewer cases are included in the study and there is the absence of long-term follow-up. Moreover, only the finished enteral nutrition preparations were used for research and analysis with the control group, and no more detailed nutrition evaluation is conducted or a detailed individualized nutrition scheme is developed. In view of this, positive countermeasures are being carried out with the progress of the study to further expand the sample size and formulate a standardized follow-up scheme, so as to further evaluate the efficacy of the treatment regimen and benefit more patients.

## CONCLUSIONS

Reasonable enteral nutrition boasts a variety of benefits for the recovery of elderly patients with chronic heart failure. With reasonable enteral nutrition, the heart function of elderly patients with chronic heart failure can be significantly improved, inflammatory factors can be reduced, immunity and performance status can be enhanced, and gastrointestinal tolerance can be improved without obvious gastrointestinal reactions.

### Authors’ Contributions:

**DZ & HL:** Designed this study, prepared this manuscript, are responsible and accountable for the accuracy or integrity of the work.

**XT:** Collected, analyzed clinical data.

**SZ:** Significantly revised this manuscript.
